# Biomolecular Investigation of *Bartonella* spp. in Wild Rodents of Two Swiss Regions

**DOI:** 10.3390/pathogens10101331

**Published:** 2021-10-15

**Authors:** Sara Divari, Marta Danelli, Paola Pregel, Giovanni Ghielmetti, Nicole Borel, Enrico Bollo

**Affiliations:** 1 Department of Veterinary Science, University of Turin, Largo Braccini 2, 10095 Turin, Italy; marta.danelli@edu.unito.it (M.D.); paola.pregel@unito.it (P.P.); enrico.bollo@unito.it (E.B.); 2 Institute for Food Safety and Hygiene, Section of Veterinary Bacteriology, Vetsuisse Faculty, University of Zurich, 8057 Zurich, Switzerland; giovanni.ghielmetti@vetbakt.uzh.ch; 3 Institute of Veterinary Pathology, Vetsuisse Faculty, University of Zurich, 8057 Zurich, Switzerland; nicole.borel@uzh.ch

**Keywords:** *Bartonella* spp., *B. taylorii*, wild rodents, Switzerland

## Abstract

Rodents represent a natural reservoir of several *Bartonella* species, including zoonotic ones. In this study, small wild rodents, collected from two sites in rural areas of Switzerland, were screened for *Bartonella* spp. using molecular detection methods. In brief, 346 rodents were trapped in two rural sites in the Gantrisch Nature Park of Switzerland (Plasselb, canton of Fribourg, and Riggisberg, canton of Bern). Pools of DNA originating from three animals were tested through a qPCR screening and an end-point PCR, amplifying the 16S-23S rRNA gene intergenic transcribed spacer region and citrate synthase (*gltA*) loci, respectively. Subsequently, DNA was extracted from spleen samples belonging to single animals of *gltA* positive pools, and *gltA* and RNA polymerase subunit beta (*rpoB*) were detected by end-point PCR. Based on PCR results and sequencing, the prevalence of infection with *Bartonella* spp. in captured rodents, was 21.10% (73/346): 31.78% in *Apodemus* sp. (41/129), 10.47% in *Arvicola scherman* (9/86), 17.05% in *Myodes glareolus* (22/129), and 50% in *Microtus agrestis* (1/2). A significant association was observed between *Bartonella* spp. infection and rodent species (*p* < 0.01) and between trapping regions and positivity to *Bartonella* spp. infection (*p* < 0.001). Similarly, prevalence of *Bartonella* DNA was higher (*p* < 0.001) in rodents trapped in woodland areas (66/257, 25.68%) compared to those captured in open fields (9/89, 10.11%). Sequencing and phylogenetic analysis demonstrated that the extracted *Bartonella* DNA belonged mainly to *B. taylorii* and also to *Candidatus* “Bartonella rudakovii”, *B. grahamii*, *B. doshiae*, and *B. birtlesii*. In conclusion, the present study could rise public health issues regarding *Bartonella* infection in rodents in Switzerland.

## 1. Introduction

*Bartonella* species are Gram-negative, facultative intracellular and emerging zoonotic bacteria infecting both domestic and wild mammals [1]. Rodents are probably the most common wildlife host of *Bartonella*, and some rodent-associated *Bartonella* spp. may induce infections in humans [2].

*Bartonella* spp. are widespread worldwide, but the prevalence is higher in areas where the climatic conditions favor spreading of the arthropod vectors [3]. Although ectoparasites (ticks, fleas, and mites) are the principal vectors allowing *Bartonella* spp. transmission among animal hosts, the ecology of these bacteria is more complex and still not well understood [4].

Human Bartonellosis can manifest with various clinical signs that are often correlated with the immune status of the subject and, obviously, with the species and bacterial load of *Bartonella* that infect the host [5].

Some *Bartonella* species lead well-known human diseases, such as *B. henselae*, responsible for cat scratch disease, and *B. quintana*, causative agent of trench fever. Others are associated with different clinical conditions such as weight loss, muscle fatigue, and neurological manifestations [4] as well as emerging diseases, including endocarditis [6], chronic lymphadenopathy, bacillary angiomatosis and peliosis, uveitis, and vasculitis [7]. *Bartonella* infection often leads to febrile illnesses and the clinical condition may be similar to those triggered by other pathogens (e.g., *Borrelia* spp.) [8]. This suggests that the diseases associated to *Bartonella* could be under-estimated. Recently, the number of newly detected *Bartonella* species increased significantly and, to date, 45 different species have been isolated [9]. These were identified in humans, domestic [10,11,12] and wild animals, including bats [13], deer [14], marine mammals [15], rodents [16], and sheep [17]. Molecular evidence of *Bartonella* spp. was reported also in some migratory bird species and sea turtles [18,19].

Rodents represent a natural reservoir of several *Bartonella* species, and different *Bartonella* spp. could infect numerous rodent species with various prevalence worldwide [20]. *B. tribocorum* and *B. elizabethae* often associated to human bartonellosis [21,22] were identified in rats. Also *B. henselae* has been identified in wild rodents, such as *Rattus rattus* from New Zealand [23], *Apodemus* spp. in Denmark [24], and in the Pianosa Island, Italy [25].

*Bartonella* spp. are slow-growing microorganisms. They need complex media as *Bartonella–Alphaproteobacteria* growth medium based on an insect growth medium and culture conditions such as 5% CO_2_, water-saturated atmosphere [26,27,28]. Moreover they are often weak reactors to many biochemical tests [29]. These characteristics hinder their isolation and identification at species level, therefore several molecular detection methods based on specific loci have been designed for the identification of *Bartonella* [28,30].

In this study, small wild rodents collected from two sites in rural areas of Switzerland were screened for *Bartonella* spp. using molecular detection methods.

## 2. Results

A total of 84/116 DNA pools (72.4%) yielded Cq values lower than 35 in the qPCR analysis for *Bartonella* spp. 16S-23S rRNA intergenic transcribed spacer (ITS). R^2^, slope, primer efficiency, and Cq mean values of qPCR were 0.998, −3.386, 97.4%, and 25.16, respectively. The subsequent conventional PCR (cPCR) identified 43 out of these 84 pools (51.2%) as also positive for the *Bartonella* spp. citrate synthase (*gltA*) locus. DNA from spleen of single animals belonging to *gltA* positive pools were then extracted, for a total of 129/346 animals. Seventy-three (56.6%) and 64 (49.6%) out of these 129 samples showed amplicons consistent with *gltA* and RNA polymerase subunit beta (*rpoB*) loci, respectively (Table S1). Based on cPCR results (Figure S1) and following sequencing, the prevalence of infection with *Bartonella* spp. in captured rodents, was 73/346 (21.10%) and a significant association (*p* < 0.01) was observed between *Bartonella* spp. infection and rodent species. Prevalence recorded for *Bartonella* spp. in *Apodemus* sp. was 31.78% (41/129): 39/129 animals tested were positive for both *gltA* and *rpoB* loci by cPCR and 2/129 positive only for *rpoB* locus. Sequencing confirmed the results. *Bartonella* DNA was detected in 9/86 (10.47%) samples of *Arvicola scherman*, and in two specimens only *gltA* was amplified. *gltA* and *rpoB* loci were detected by cPCR in 24 out of 129 *Myodes glareolus*, but two of them were not confirmed by sequencing: therefore, the prevalence of *Bartonella* spp. in this rodent species was 17.05% (22/129); in details, 3/22 animals were positive only to *gltA*. Moreover, *gltA* locus amplification was observed in 1/2 (50%) samples of *Microtus agrestis*.

Prevalence of *Bartonella* DNA identified in the rodents captured in the two municipalities was 30.71% (43/140) in Riggisberg (BE) and 15.05% (31/206) in Plasselb (FR), and a significant association (*p* < 0.001) between trapping region and positivity to *Bartonella* spp. was observed. Similarly, prevalence of *Bartonella* DNA was significantly higher (*p* < 0.001) in rodents trapped in woodland areas (66/257, 25.68%) compared to those captured in open fields (9/89, 10.11%). No statistically significant association between *Bartonella* DNA presence and gender or age of captured rodents was observed.

**Figure 1:**
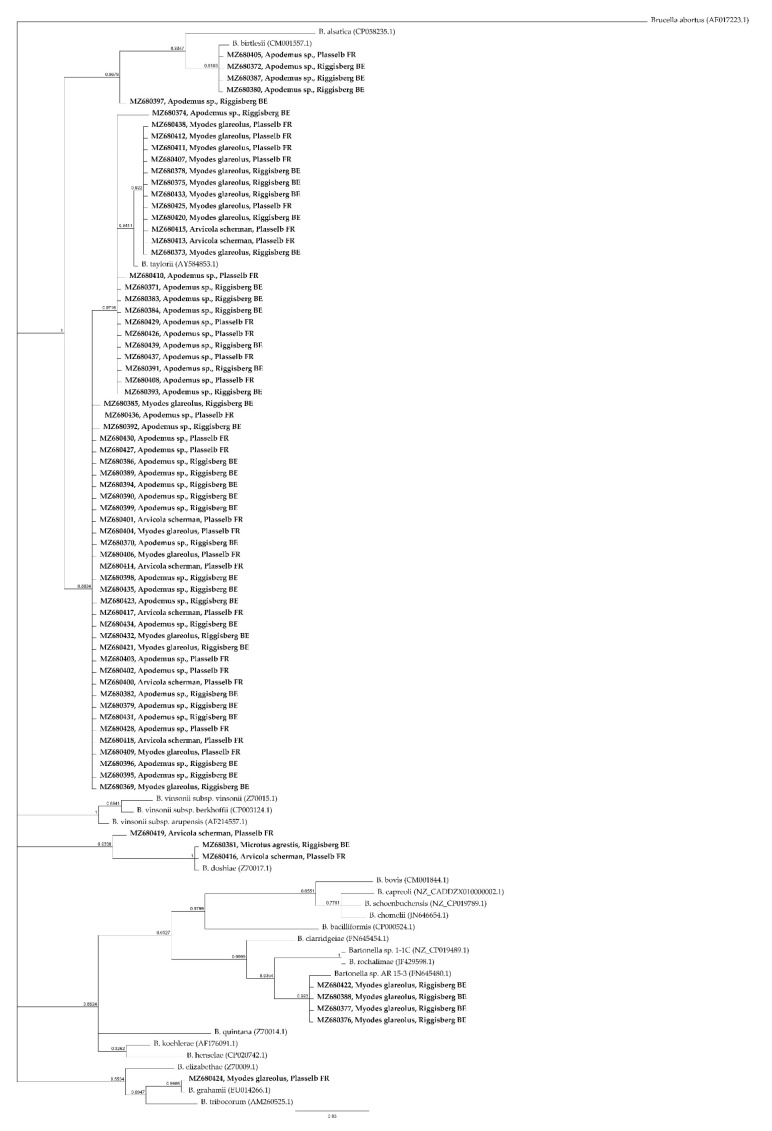
Phylogenetic tree based on the *gltA* (293 bp) partial sequences of *Bartonella* spp. Sequences identified in the present study are indicated in bold (GenBank accession number, host, and site of trapping) and sequences from GenBank are indicated as common name and GenBank accession number in bracket. The phylogenetic tree was constructed using Bayesian inference method, using a GTR substitution model with the MrBayes plugin in Geneious Prime version 2021.2.2, with 1,100,000 chain length, 100,000 burn-in length. *Brucella abortus* was used as outgroup.

**Figure 2:**
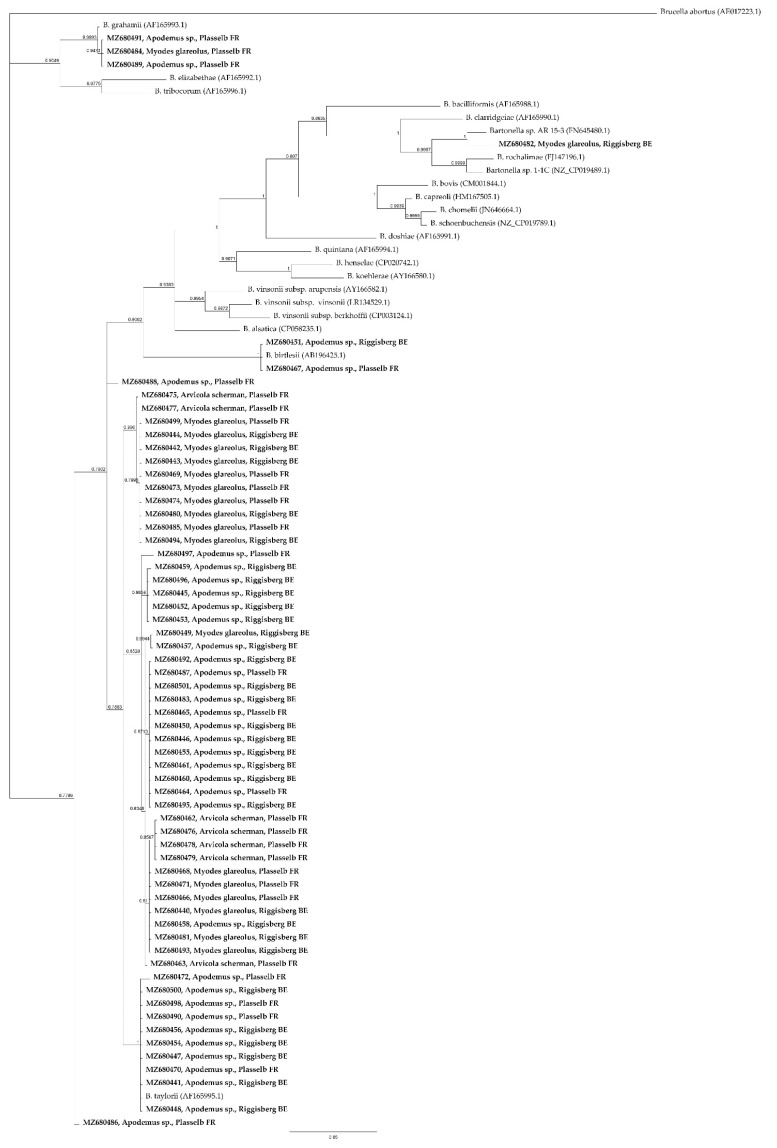
Phylogenetic tree based on the *rpoB* (682 bp) partial sequences of *Bartonella* spp. Sequences identified in the present study are indicated in bold (GenBank accession number, host, and site of trapping) and sequences from GenBank are indicated as common name and GenBank accession number in bracket. The phylogenetic tree was constructed using Bayesian inference method with the MrBayes plugin in Geneious Prime version 2021.2.2, with 1,100,000 chain length, 100,000 burn-in length. *Brucella abortus* was used as outgroup.

*GltA* sequences, amplified by cPCR, showed 100% identity to *Candidatus* “Bartonella rudakovii” (EF682090.1) in four *Myodes glareolus* out of 129 animals (3.10%). The *gltA* amplicon sequence in one *Myodes glareolus* out of 129 (0.78%) was 100% similar to *B. grahamii* (CP001562.1). Two *Bartonella gltA* sequence detected in *Arvicola scherman* (1/86, 1.16%) and *Microtus agrestis* (1/2, 50%) were 100% identical to *B. doshiae* (Z70017.1). *B. taylorii* (AF165995.1) was identified with 100% of identity sequencing *rpoB* amplicons (95–98% of query cover) in eight *Apodemus* sp. samples out of 129 (6.2%) and in two *Apodemus* sp out of 129 (1.6%) was identified the *rpoB* sequence of *B. birtlesii* (AB196425.1) (100% of identity). In one *Apodemus* sp. *B. taylorii* (AF165995.1, 98.15% of identity) and *B. grahamii* (CP001562.1, 99.65% of identity) DNA were identified by *gltA* and *rpoB* amplicons sequencing respectively. The remaining *Bartonella* positive animals showed a *gltA* and *rpoB* sequence identity > 96.0% and > 95.4%, respectively to closest relatives present in GenBank. In particular, considering the criteria previously established by La Scola et al. [30], *B. taylorii* was identified in 59/346 animals. Detailed results are shown in Supplementary Materials (Table S1). A BLASTn and phylogenetic analysis of *gltA* (Figure 1) and *rpoB* (Figure 2) loci identified in this study revealed that most of *Bartonella* DNA isolated are closely related to *B. taylorii*, followed by *B. grahamii*, *B. birtlesii*, and *B. doshiae*.

## 3. Discussion

In this study the prevalence and molecular diversity of *Bartonella* in small rodent populations from Switzerland were firstly described.

Wild rodents could be potential reservoirs causing *Bartonella* infections and more than 20 *Bartonella* species are associated with these small mammals [31]. They include some zoonotic species, such as *B. elizabethae*, *B. grahamii*, and *B. vinsonii* subsp. *arupensis* [20,32].

In central Europe, prevalences of *Bartonella* spp. ranging from 3.3 to 65.8% [33] have been observed in wild rodents. The prevalence of about 21% reported in our study was similar to the ones observed in Lithuania (24%) in 2013–2014 period [34] and Poland (11–48%) [20]. In particular, *B. taylori*-like DNA was the most common detected species in this study, followed by *B. grahamii* and *B. birtlesii*, three of the four most widespread species in European rodents [33].

Phylogenetic analysis based on *gltA* and *rpoB* loci demonstrated that in wild rodents of Switzerland multiple *Bartonella* species were identified and four genogroups were recognized, in particular *B. grahamii*, *B. taylorii*, *B. doshiae* and *B. birtlesii*. [20]. According to the lineages previously specified by Engel et al. [35], *Bartonella* species detected in the present study belong to lineage three and four.

*B. taylorii* was isolated for the first time by Birtles et al. [36]. DNA of *B. taylorii* strain Far East II was previously identified in rodents including *Apodemus agrarius* from Russian Far East in 2005 [37] and also in the present study. In Europe, *B. taylorii* was principally detected in *Myodes* sp. and *Microtus* sp. [38].

In this study, *Bartonella* DNA relative to *B. grahamii* was identified in a *Myodes glareolus*, and it is known that this bacteria may cause neuroretinitis in humans [39]. In one *Apodemus* sp. a probable co-infection of *B. grahamii* with *B. taylorii* was observed, confirming the wide range of hosts and the worldwide distribution of *B. grahamii*-like organisms, as described in Szewczyk et al., [33]. Buffet et al. [38] reported the presence of *B. grahamii* in *Microtus* spp. and *Apodemus* spp. in France.

*Bartonella* sequences identified in four *Myodes glareolus* trapped in the municipality of Riggisberg were 100% identical to *Candidatus* “Bartonella rudakovii” identified in 2007 in small wild mammals in Western Siberia (unpublished, GenBank: EF682090.1).

*RpoB* sequences of three *Apodemus* sp. captured in the present project were very close to *B. birtlesii*, isolated for the first time in small rodents in Germany and France [40].

From three animals, DNA similar to *gltA* of *B. doshiae* was identified (94.82–100% of identity). This species was reported in mice and voles in Europe [41] and in *Sigmodon hispidus* in the United States [42]. Vayssier-Taussat et al. [43] highlighted their novel potential zoonotic properties.

In this study, *Apodemus* sp. was the rodent genus more frequently affected by *Bartonella* sp. Paziewska et al. [44] obtained similar results, showing that *A. flavicollis* was the species in which *Bartonella* sp. was more present in Poland. Moreover, as in Poland, the present findings showed that *B. taylorii* was the most common *Bartonella* species, with a higher prevalence in *Apodemus* than in *Myodes*. Similarly, in 2019, a new study on the presence of *Bartonella* spp. in rodents was conducted in the Baltic region. The prevalence of *Bartonella* spp. was 54.8% and, in particular, *A. flavicollis* and *M. agrestis* were the most infected rodent species [45].

In this study, rodents trapped in woodland areas were more often infected with *Bartonella* spp. compared to those captured in open fields. A possible explanation for this phenomenon is the presence of vectors, such as fleas, which prefer wet conditions of woodland, allowing a better survival of larval stages [46]. In fact, ectoparasites are influenced by host characteristics, host environment, and season [46,47,48]. However, temperature and humidity conditions can affect the various vector species differently, therefore further investigations on parasites present in the considered geographical area would be useful. The distribution of reservoirs or arthropod vectors are likely the reasons why the prevalence of *Bartonella* infection varied among different rodent species or locations.

## 4. Materials and Methods

Wild rodents studied were trapped between April and November 2017 in the Gantrisch Nature Park (Switzerland) as described in Peterhans et al. [49], in the municipalities of Plasselb (canton of Fribourg) and Riggisberg (canton of Bern).

Topcat traps (Andermatt Biocontrol, Switzerland) were used in open fields, whereas live traps (Longworth, Penlon Ltd., Abingdon, UK) were placed in the woodland.

Trapped mice were visually examined and then euthanized by exposure to carbon dioxide on site. Mice were then transported refrigerated to the laboratory, where postmortem examination and sampling were performed as previous described in Peterhans et al. [49] (Table 1). The age of *Arvicola* was determined by measuring the weight of dry crystalline lenses [50,51]. For the remaining species, the development of the sexual organs was used as in Beerli et al. [52].

This project was performed in accordance with the Swiss Animal Welfare Act (SR 455) and the regulations of the Cantons of Bern and Fribourg (permit number BE145/16).

Lungs, spleen, liver, mandibular, and mesenteric lymph nodes were collected from 346 animals, and tissue samples of three animals were pooled, resulting in a total of 116 pools. These were homogenized and genomic DNA was obtained using DNeasy Blood and Tissue Kit (Qiagen, Hilden, Germany). A real-time PCR was performed on the pools to detect *Bartonella* spp. DNA as described in Divari et al. [25]. ITS region of *Bartonella* spp. was amplified (about 200 bp) using published primers 321s and H493as [53], [26] and amplification was performed using the CFX ConnectTM Real-Time PCR Detection System (BioRad, Hercules, CA, USA). In brief, iTaq Universal SYBER^®^ Green Supermix (BioRad, Hercules, CA, USA) was used in the reaction mix and the protocol consisted of a 4-min step at 94 °C, followed by 45 cycles at 95 °C for 5 s and 60 °C for 20 s. A melting curve (from 65 °C to 95 °C) was obtained at the end of each run, to detect the PCR products dissociation. Examples of Tm values were described in Supplementary Materials (Table S2). The efficiency of qPCR was calculated on the slope of the standard curve constructed for ITS region amplification using scalar dilution of DNA from the positive control (*Bartonella* sp. FG4-1).

Pools showing a quantification cycle (Cq) less than 35 were further analyzed by cPCR, detecting a 340 bp segment of the *gltA* specific for *Bartonella* spp. cPCR was performed using previously described primers 443f and 781r [25,54] and a master mix (HotStarTaq; Qiagen, Hilden, Germany). The *gltA* gene fragment was amplified by a protocol consisting of a first step at 95 °C for 15 min, followed by 45 cycles at 95 °C for 5 s and 60 °C for 45 s.

PCR products were electrophoresed in 1.5% and 2% agarose gels, stained by GelRed Nucleic Acid Gel Stain (Biotium Inc., Fremont, CA, USA) and visualized under UV light. *Bartonella* spp. FG4-1 DNA (NCBI: txid545598) was used as a positive control and nuclease-free water included as a negative control in each PCR run. Pools showing amplicons consistent in size with the amplified locus were considered positive to *Bartonella* spp. infection. Therefore, DNA from spleen of single animals belonging to positive pools was extracted using DNeasy Blood and Tissue Kit (Qiagen, Hilden, Germany) and retested for *gltA* (as described above) and *rpoB* loci. For this latest gene, an 800 bp segment was amplified using previously described primers 1400f and 2300r [23,55] and amplification was conducted under the following conditions: 95 °C for 15 min, followed by 35 cycles of denaturation at 95 °C for 30 s, annealing at 53 °C, and extension at 72 °C for 1 min. At the end of the reaction, an additional extension step at 72 °C for 2 min was applied.

PCR products were visualized by electrophoresis, purified through MinElute PCR purification kit (Qiagen) and sequenced in both directions using Sanger method by a commercial sequencing provider (BMR Genomics, Padova, Italy).

The raw sequences were edited using Geneious Prime version 2021.2.2 [56] and compared to sequences deposited in NCBI using BLAST (https://blast.ncbi.nlm.nih.gov/Blast.cgi (accessed on 2 August 2021)). All sequences were deposited in the GenBank under accession numbers described in Supplementary Materials (Table S3).

*GltA* and *rpoB* sequences from this study and from GenBank were aligned (MUSCLE alignment algorithm) and phylogenetic relations were estimated through a Bayesian inference method using a GTR substitution model with the MrBayes [57] plugin in Geneious Prime version 2021.2.2 [56], with 1,100,000 chain length, 100,000 burn-in length.

Differences of *Bartonella* spp. prevalence among small rodent species, sampling locations, gender and age were assessed by Fisher’s exact and Chi-square test and 95% confidence intervals were set. Statistical analysis was conducted using GraphPad Prism 6 version 6.07 and a *p* value < 0.05 was considered significant.

## 5. Conclusions

Wild rodents infected with zoonotic *Bartonella* species were detected in two rural areas of Switzerland. However, these regions are not distant from urban areas and, thus, contacts between humans and infected rodents are possible. Therefore, the *Bartonella*-infected wild rodents might represent a potential pathogen reservoir in Switzerland and should be considered of public health importance.

## Figures and Tables

**Table 1:** Origin of trapping (BE: Riggisberg; FR: Plasselb; WL: woodland; OF: open field), species, gender, age, and number of wild rodents analyzed.

Rodent Species	Site		Area		Gender		Age		Total
	BE	FR	WL	OF	Females	Males	Adult	Juvenile	
*Myodes glareolus*	55	74	128	1	66	63	84	45	129
*Microtus agrestis*	2	0	2	0	2	0	2	0	2
*Arvicola scherman*	0	86	0	86	47	39	69	17	86
*Apodemus* sp.	83	46	127	2	63	66	90	39	129

## Data Availability

The sequences obtained in the current study were submitted to GenBank under the accession numbers MZ680369 to MZ680501.

## Supplementary Materials

The following tables are available online at https://www.mdpi.com/article/10.3390/pathogens10101331/s1, Table S1: Sequencing results of *Bartonella* spp. positive rodents. Table S2: examples of Tm values calculated for DNA of samples analysed by qPCR. Table S3: Accession number of sequences deposited in GenBank. Figure S1: examples of cPCR products, relative to *gltA* and *rpoB* loci amplification.
