# Supplementary material for: Biomolecular Investigation of Bartonella spp. in Wild Rodents of Two Swiss Regions
Source: Pathogens. 2021 Oct 15;10(10):1331. doi: 10.3390/pathogens10101331 (PMC8539893; doi:10.3390/pathogens10101331)
Supplement: Supplementary file 1 [file pathogens-10-01331-s001.zip › pathogens-1351863-supplementary.pdf]

pathogens

MDPI

Supplementary Materials

Biomolecular Investigation of *Bartonella* spp. in Wild Rodents of Two Swiss Regions

Sara Divari [1,*], Marta Danelli [1], Paola Pregel [1], Giovanni Ghielmetti [2], Nicole Borel [3], and Enrico Bollo [1]

[1] Department of Veterinary Science, University of Turin, Largo Braccini 2, 10095 Turin, Italy; marta.danelli@edu.unito.it (M.D.); paola.pregel@unito.it (P.P.); enrico.bollo@unito.it (E.B.)
[2] Institute for Food Safety and Hygiene, Section of Veterinary Bacteriology, Vetsuisse Faculty, University of Zurich, 8057 Zurich, Switzerland; giovanni.ghielmetti@vetbakt.uzh.ch
[3] Institute of Veterinary Pathology, Vetsuisse Faculty, University of Zurich, 8057 Zurich, Switzerland; nicole.borel@uzh.ch
* Correspondence: sara.divari@unito.it

Citation: Divari, S.; Danelli, M.; Pregel, P.; Ghielmetti, G.; Borel, N.; and Bollo, E. Biomolecular Investigation of *Bartonella* spp. in Wild Rodents of Two Swiss Regions. *Pathogens* **2021**, *10*, 1331. https://doi.org/10.3390/pathogens10101331

Academic Editor: Edward B. Breitschwerdt

Received: 6 August 2021
Accepted: 11 October 2021
Published: 15 October 2021

Publisher's Note: MDPI stays neutral with regard to jurisdictional claims in published maps and institutional affiliations.

Copyright: © 2021 by the authors. Licensee MDPI, Basel, Switzerland. This article is an open access article distributed under the terms and conditions of the Creative Commons Attribution (CC BY) license (http://creativecommons.org/licenses/by/4.0/).

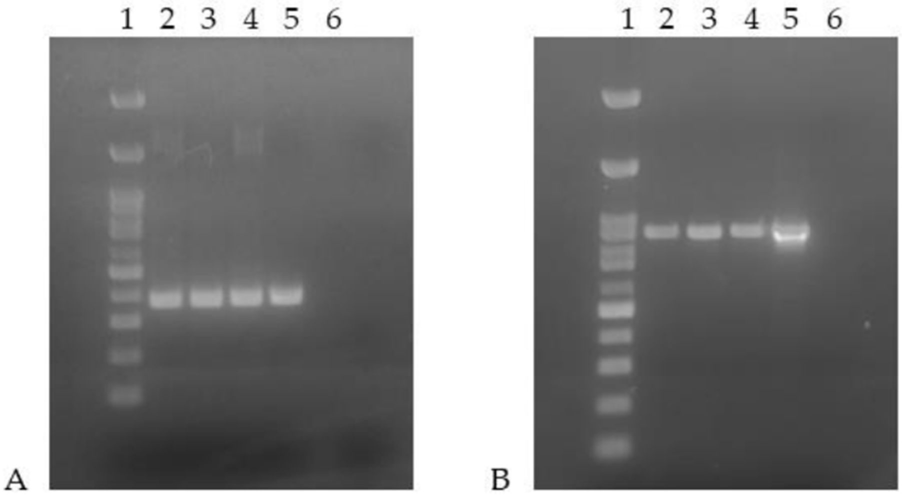

Figure S1. Example of gel electrophoresis for gltA (about 340 bp) (A) and rpoB (about 800 bp) PCR amplification (B) of three Bartonella positive samples and a positive control (DNA from Bartonella sp. FG4-1). Lane 1, 100 bp DNA ladder (Nippon Genetics Europe, Düren, Germany); lane 2, sample M127; lane 3, sample M133; lane 4, sample M302; lanes 5, positive control; lane 6, no template control.

Table S1. Sequencing results of *Bartonella* spp. positive rodents, including the closest relative sequence (query cover 99–100%, E value 0–2,00E-180 for *gltA* and 94-100%, E value 0 for *rpoB* sequences) and identity results (ID %) for *gltA* and *rpoB* loci, number (n.), rodent species infected, and origin of trapping.

N. Sample	Rodent Species	ID Sample	Origin	DNA Sequences Closest Relative (Accession No.)			
				gltA	ID %	*rpoB*	ID %
10	*Apodemus* sp.	M126	Plasselb FR	*Bartonella taylorii* (AY584853.1)	98.58	*Bartonella taylorii* (AF165995.1)	97.06
		M127			98.58		97.50
		M172			99.15		100.00
		M181			98.59		98.98
		M263			99.15		95.88
		M265			97.81		97.12
		M266			98.34		96.84
		M270			99.15		100.00
		M301			98.11		97.03
		M302			99.15		100.00
1	*Apodemus* sp.	M278	Plasselb FR	*Bartonella taylorii* (AY584853.1)	98.15	*Bartonella grahamii* as4aup (CP001562.1)	99.65
21	*Apodemus* sp.	M19	Riggisberg BE	*Bartonella taylorii* (AY584853.1)	99.15	*Bartonella taylorii* (AF165995.1)	100.00
		M40			98.58		97.11
		M50			98.58		97.09
		M52			99.16		100.00
		M53			99.16		100.00
		M66			98.56		97.09
		M69			98.59		97.25
		M70			98.59		97.03
		M71			99.15		99.88
		M72			98.29		97.09
		M74			99.15		100.00
		M75			98.59		97.06
		M76			98.57		97.21
		M77			98.56		97.21
		M79			98.58		97.09
		M80			98.59		97.09
		M247			98.58		97.09

		M279			98.37		97.09
		M294			98.55		97.09
		M296			98.58		97.21
		M340			99.15		100.00
		M6			98.58	No amplicon	
3	*Apodemus* sp.	M78	Riggisberg BE	*Bartonella taylorii* (AY584853.1)	94.03	No amplicon	
		M30			96.62	No amplicon	
		M107			98.58		96.97
		M112			98.56		97.21
		M187			99.44		97.33
7	*Arvicola sherman*	M188	Plasselb FR	*Bartonella taylorii* (AY584853.1)	98.58	*Bartonella taylorii* (AF165995.1)	96.97
		M189			99.44		97.33
		M203			98.58		96.97
		M220			98.58		96.97
		M129			98.58		97.19
		M135			98.57		97.21
		M142			99.72		97.33
8	*Myodes glareolus*	M174	Plasselb FR	*Bartonella taylorii* (AY584853.1)	98.57	*Bartonella taylorii* (AF165995.1)	97.21
		M182			99.72		97.33
		M183			99.72		97.33
		M262			99.44		97.94
		M303			99.72		97.33
		M3			98.31		97.27
		M28			99.71		97.33
		M32			99.45		97.33
		M36			99.72		97.33
9	*Myodes glareolus*	M54	Riggisberg BE	*Bartonella taylorii* (AY584853.1)	98.01	*Bartonella taylorii* (AF165995.1)	97.28
		M244			99.72		97.33
		M245			98.29		97.21
		M292			98.58		97.21
		M293			99.71		97.33
1	*Microtus agrestis*	M42	Riggisberg BE	*Bartonella doshiae* (Z70017.1)	100	No amplicon	
2	*Arvicola sherman*	M202	Plasselb FR	*Bartonella doshiae* (Z70017.1)	100	No amplicon	

		M221			94.82	No amplicon	
1	*Myodes glareolus*	M261	Plasselb FR	*Bartonella grahamii* as4aup (CP001562.1)	100	*Bartonella grahamii* as4aup (CP001562.1)	99.88
2	*Apodemus* sp.	M21	Riggisberg BE	*Bartonella* sp. (Z70012.1)	98.59	No amplicon	
		M41			98.60	Very low quality sequence	
1	*Apodemus* sp.	M67	Riggisberg BE	*Bartonella* sp. (Z70012.1)	98.48	*Bartonella birtlesii* (AB196425.1)	100.00
1	*Apodemus* sp.	M133	Plasselb FR	*Bartonella* sp. (Z70012.1)	98.59	*Bartonella birtlesii* (AB196425.1)	100.00
3	*Myodes glareolus*	M33	Riggisberg BE	*Candidatus* Bartonella rudakovii (EF682090.1)	100	No amplicon	
		M35			100	No amplicon	
		M68			100	No amplicon	
1	*Myodes glareolus*	M246	Riggisberg BE	*Candidatus* Bartonella rudakovii (EF682090.1)	100	*Candidatus* Bartonella rudakovii (EF682088.1)	99.87
1	*Myodes glareolus*	M31	Riggisberg BE	Very low quality sequence		No amplicon	
1	*Apodemus* sp.	M342	Riggisberg BE	Aspecific sequence		*Bartonella taylorii* (AF165995.1)	97.12
1	*Apodemus* sp.	M267	Plasselb FR	No amplicon		*Bartonella grahamii* as4aup (CP001562.1)	99.65
1	*Myodes glareolus*	M297	Riggisberg BE	No amplicon		Very low quality sequence	

ND: not detectable

Table S2. Melting temperature (Tm) calculated for DNA of the positive control (*Bartonella* sp. FG4-1) and of three positive samples analysed by qPCR for ITS region of *Bartonella* spp. (about 200 bp).

ID Animals	Tm (°C) Mean ± Standard Deviation
FG4-1	81.00 ± 0.00
M127	81.55 ± 0.16
M133	80.50 ± 0.53
M302	81.30 ± 0.35

Table S3. Accession number of sequences deposited in GenBank.

ID samples	Accession numbers	
	gltA	*rpoB*
M3	MZ680369	MZ680440
M6	MZ680370	
M19	MZ680371	MZ680441
M21	MZ680372	
M28	MZ680373	MZ680442
M30	MZ680374	
M32	MZ680375	MZ680443
M33	MZ680376	
M35	MZ680377	
M36	MZ680378	MZ680444
M40	MZ680379	MZ680445
M41	MZ680380	
M42	MZ680381	
M50	MZ680382	MZ680446
M52	MZ680383	MZ680447
M53	MZ680384	MZ680448
M54	MZ680385	MZ680449
M66	MZ680386	MZ680450
M67	MZ680387	MZ680451
M68	MZ680388	
M69	MZ680389	MZ680452
M70	MZ680390	MZ680453
M71	MZ680391	MZ680454
M72	MZ680392	MZ680455
M74	MZ680393	MZ680456
M75	MZ680394	MZ680457
M76	MZ680395	MZ680458
M77	MZ680396	MZ680459
M78	MZ680397	
M79	MZ680398	MZ680460
M80	MZ680399	MZ680461
M107	MZ680400	MZ680462
M112	MZ680401	MZ680463
M126	MZ680402	MZ680464
M127	MZ680403	MZ680465
M129	MZ680404	MZ680466
M133	MZ680405	MZ680467
M135	MZ680406	MZ680468
M142	MZ680407	MZ680469
M172	MZ680408	MZ680470

M174	MZ680409	MZ680471
M181	MZ680410	MZ680472
M182	MZ680411	MZ680473
M183	MZ680412	MZ680474
M187	MZ680413	MZ680475
M188	MZ680414	MZ680476
M189	MZ680415	MZ680477
M202	MZ680416	
M203	MZ680417	MZ680478
M220	MZ680418	MZ680479
M221	MZ680419	
M244	MZ680420	MZ680480
M245	MZ680421	MZ680481
M246	MZ680422	MZ680482
M247	MZ680423	MZ680483
M261	MZ680424	MZ680484
M262	MZ680425	MZ680485
M263	MZ680426	MZ680486
M265	MZ680427	MZ680487
M266	MZ680428	MZ680488
M267		MZ680489
M270	MZ680429	MZ680490
M278	MZ680430	MZ680491
M279	MZ680431	MZ680492
M292	MZ680432	MZ680493
M293	MZ680433	MZ680494
M294	MZ680434	MZ680495
M296	MZ680435	MZ680496
M301	MZ680436	MZ680497
M302	MZ680437	MZ680498
M303	MZ680438	MZ680499
M340	MZ680439	MZ680500
M342		MZ680501
